# Papillary fibroelastoma of the left atrial wall: a case report

**DOI:** 10.1186/1749-8090-4-28

**Published:** 2009-07-01

**Authors:** Murat Bicer, Mustafa Cikirikcioglu, Erman Pektok, Hajo Müller, Sarah Dettwiler, Afksendiyos Kalangos

**Affiliations:** 1 Department of Cardiovascular Surgery, University Hospital and Medical Faculty of Geneva, Geneva, Switzerland; 2 Department of Cardiology, University Hospital and Medical Faculty of Geneva, Geneva, Switzerland; 3 Department of Clinical Pathology, University Hospital and Medical Faculty of Geneva, Geneva, Switzerland

## Abstract

Cardiac papillary fibroelastoma is a rare, benign cardiac tumor. It often arises from valvular endocardium, and non-valvular endocardial location is rare. Although transthoracic echocardiography is usually sufficient for the diagnosis of most cardiac tumors, small tumors such as papillary fibroelastoma may be missed. Transesophageal echocardiography is superior to transthoracic echocardiography in diagnosing these tumors. Despite their benign histology, and independent of their size, they should be resected surgically because of their high potential for embolization.

In this report, we present a case of papillary fibroelastoma located on the left atrial wall, presenting with symptoms of cerebral ischemia. The patient was treated surgically for the prevention of further embolic complications. Pertinent literature is also reviewed for this rare and benign cardiac tumor.

## Introduction

Cardiac papillary fibroelastoma (PFE) is a rare, benign cardiac tumor. Usually, it arises from valvular endocardium. Nonvalvular endocardial location is rare, and may confuse the clinician for the differential diagnosis between organized mobile thrombus, pedinculated myxoma and fibroelastoma [1-3]. Herein, we present a case of PFE presenting with symptoms of cerebral ischemia. The pertinent literature is also reviewed for this rare and benign cardiac tumor.

## Case report

**Figure 1:**
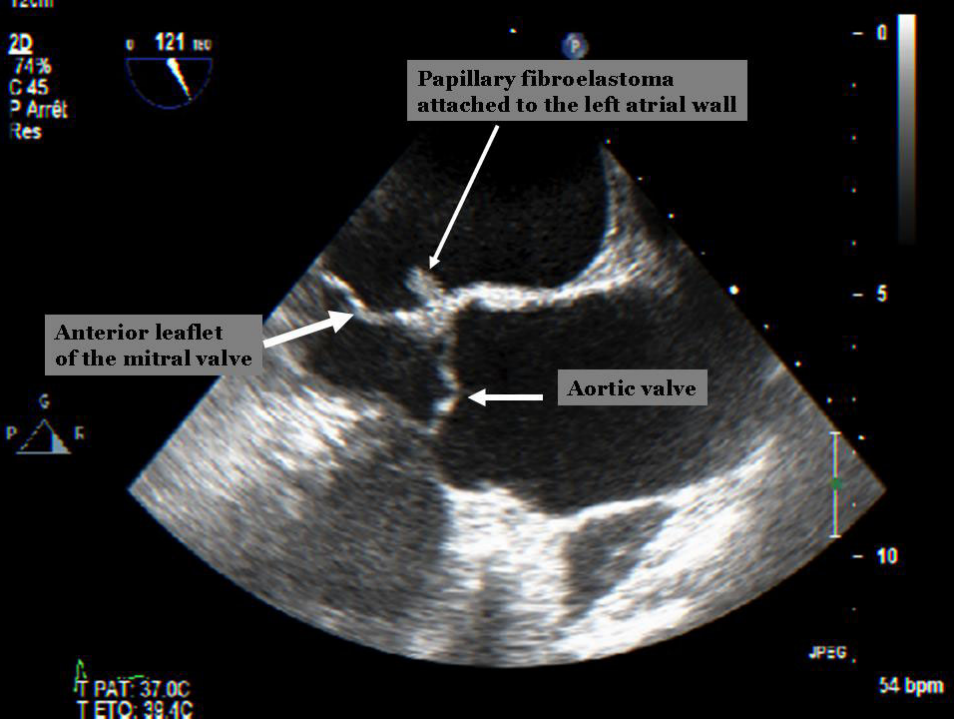
**Pre-operative trans-esophageal echocardiographic image showing small mobile mass attached to the left atrial wall on the level of the aortic valve**.

A seventy-two year old man was hospitalized in the Department of Neurology for the treatment and investigation of etiology for his ischemic cerebral event. Physical examination was unremarkable except monoparesis of the right upper extremity, right fascial paralysis and a pulsatile abdominal mass. Transthoracic echocardiography (TTE) showed a 0.7 × 0.7 cm mobile mass attached to the left atrial wall at the base of the anterior mitral leaflet. A transesophageal echocardiography (TEE) was performed for the differential diagnosis, and revealed a mass measuring 1.2 × 0.8 cm, which was attached to the left atrial wall at the level of the aortic non-coronary leaflet. Pre-operative diagnosis according to TEE was pedinculated left atrial myxoma (Figure 1). An infra-renal abdominal aortic aneurysm was also diagnosed after a thoracoabdominal computed tomography. The patient was scheduled for surgical treatment 6 weeks after his ischemic neurologic event.

**Figure 2:**
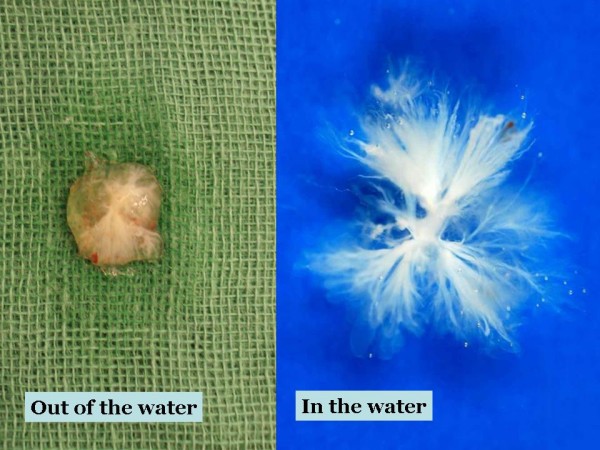
**Macroscopic images of the resected tumor in and out of water**. The anemon-like appearance is classic for papillary fibroelastomae, which looks like a solid tumor out of water.

The operation was performed under normothermic cardiopulmonary bypass using ascending aortic and bicaval cannulation. After cardiac arrest with antegrade cardioplegia, left atrium was opened by extended vertical transatrial septal (Guiraudon) incision for optimal surgical exposure. A 1-cm, gelatinous and solid looking mass (Figure 2) was found attached to the left atrial wall near the posteromedial mitral commisure. It was resected with its stalk, and the fenestration was directly closed. The resected mass changed its shape in water to an arboreous, plushy and sea-anemon like tumor. Per-operative TEE confirmed normal valvular functions and absence of residual left atrial mass.

**Figure 3:**
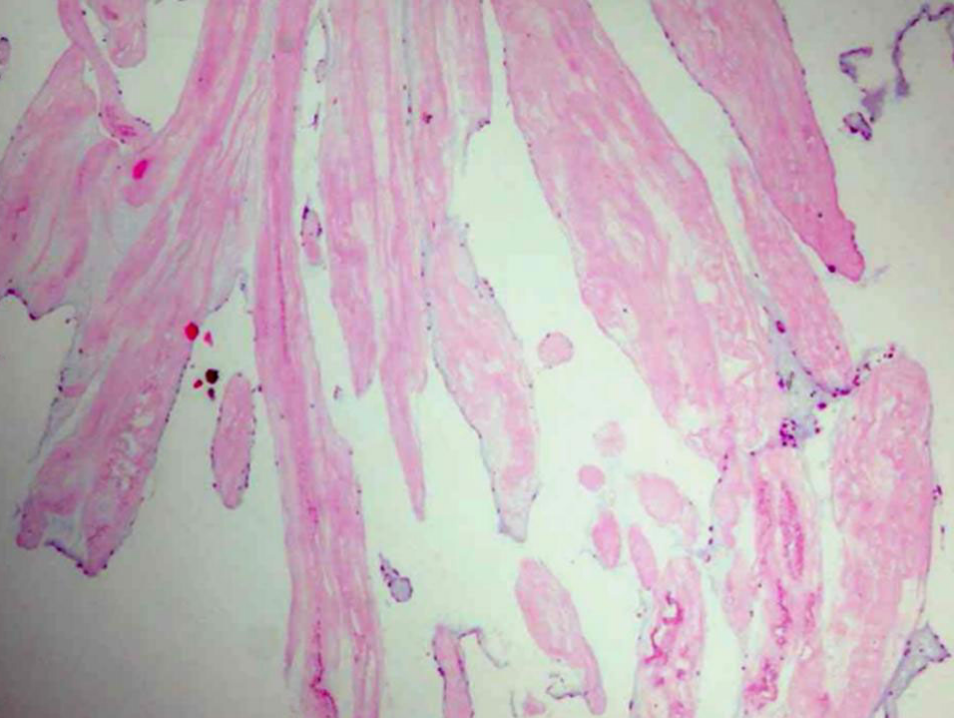
**Haematoxylin-eosin staining shows hyalinised collagenous matrix encountered by endothelial cells**.

Histologic examination of the resected tumor revealed a papillary proliferation including few fibroblasts and collagenous tissue, covered with endothelial cells (Figure 3). These morphologic and histologic findings warranted the diagnosis of PFE. The early postoperative period was uncomplicated, and the patient was discharged on postoperative day-10.

## Discussion

The incidence of cardiac tumors in autopsy series is estimated at 0.021%, and cardiac PFE constitutes 10% of these [4]. It is the third most frequent primary cardiac tumor, after myxoma and fibroma [1-3], and the most common primary tumor of heart valves. They are often found on aortic and mitral valves, less frequently on tricuspid and pulmonary valves, and rarely along atrial or ventricular walls [5-9].

The histogenesis of PFE is still unclear [3,6,8]. There are several hypotheses about the etiology. They have been considered as neoplasms, hamartomas, organized thrombi, and unusual endocardial responses to infection or hemodynamic trauma [3,8]. Kurup et al. reported that thoracic irradiation and open cardiac surgery might be the potential causes for this pathology [3]. On the other hand, histochemical presence of fibrin, hyaluronic acid, and laminated elastic fibers supports the hypothesis that PFE may be related to organizing thrombi [8,10]. A recent study proposed that it may be related to a chronic form of viral endocarditis, based on the presence of dendritic cells and cytomegalovirus in some patients [11].

The location of PFE in the heart is very important because of its potential to embolize. Left-sided tumors may cause stroke, myocardial infarction, mesenteric ischemia, renal infarction and limb ischemia. Depending on their size and mobility, PFE can also give rise to obstruction of left ventricular filling during diastole, resulting in recurrent pulmonary edema. Right-sided cardiac tumors remain predominantly asymptomatic until they become large enough to interfere with intracardiac blood flow, alter hemodynamic function or induce arrhythmias. These features can mimic the clinical picture of tricuspid valve stenosis [8]. Chronic, repeating pulmonary embolization may lead to significant hypoxemia and severe pulmonary hypertension [12,13]. Other symptoms are dyspnea, chest discomfort, and palpitations.

The diagnosis is usually made by echocardiography. Although TTE is sufficient for the diagnosis of most cardiac tumors, small tumors such as PFE may be missed, as evidenced by the present case. TEE is superior to TTE in diagnosing these tumors [2,9]. Echocardiographic features of PFE include; 1. Small lesions, typically less than 1 cm in diameter, but may be as large as 3 to 4 cm; 2. Highly mobile mass with a pedicle or stalk attached to the valve or endocardium; and 3. Frond-like appearance [5]. Recently, more cases diagnosed by magnetic resonance imaging and multislice spiral computed tomography have been reported [14,15].

There is still a debate for surgical treatment of asymptomatic patients. Despite their benign histology, surgical excision is mandated regardless of the size if the patient has recurrent embolic complications. If the patient has cerebral embolisation, the operation should be delayed at least 4 weeks in order to prevent hemorrhagic transformation of the ischemic infarct. Several atrial incisions might be used for surgical exposure. We prefer trans-septal or extended vertical trans-atrial septal (Guiraudon) incisions for complete resection of left atrial tumors with optimal exposure, if the patient does not have dilated left atrium. Differential diagnosis of PFE encompasses other heart tumors, thrombi, vegetations, valvular calcification and Lambl's excrescences. Despite its typical shape, imaging techniques may fail to differentiate PFE from other cardiac tumors, as evidenced in this case. Histological investigation after surgical resection is mandatory to confirm the diagnosis. Cardiac myxoma is a predominant left atrial tumor, and is usually attached to the atrial septum by a stalk. Histologically, myxoma differs from PFE by the presence of polygonal myxoma cells and blood vessels within the papillae. Cardiac fibroma frequently demonstrates calcification and cystic degeneration. Cardiac rhabdomyomae are predominant in infants and children. Metastic tumors of the heart are more frequent than primary tumors [4]. Unlike PFE, malignant tumors commonly involve the pericardium and myocardium, and are usually accompanied by systemic symptoms. However, with both primary and metastatic tumors, the clinical course may be complicated by emboli.

In conclusion, PFE are histologically benign tumors of the heart. They have the potential for peripheral or pulmonary embolisation regardless of their size. Since it is usually small, it cannot be detected reliably by TTE, thus TEE should be considered for a patient with an unexplained neurological ischemic event. Although there is a debate for resection of the asymptomatic PFE, surgical excision is mandated regardless of the size in order to hinder future embolic and hemodynamic complications.

## Declaration of conflict of interests

The authors declare that they have no competing interests.

## Authors' contributions

MB assisted the operation and participated manuscript writing. MC assisted the operation and participated manuscript writing. EP participated manuscript writing. HM performed echocardiographic examinations. SD made morphologic and histo-pathologic examination of the resected tumor. AK is the surgeon and participated manuscript writing. All authors read and approved the final manuscript.

## Consent section

Written informed consent was obtained from the patient for publication of this case report and accompanying images. A copy of the written consent is available for review by the Editor-in-Chief of this journal.
